# From bedside to seaside: An academic's attempt at freediving

**DOI:** 10.1113/EP092405

**Published:** 2025-02-19

**Authors:** Anthony R. Bain

**Affiliations:** ^1^ Department of Kinesiology University of Windsor Windsor Ontario Canada

Early into my PhD at the University of British Columbia, my supervisor, Prof. Philip Ainslie, asked me to join a 2‐week research trip to Split, Croatia. The answer was of course yes. The purpose of the trip was still unclear to me, but it didn't matter, as the prospect of a paid overseas research adventure was reason enough.

I was later informed that we would be studying elite freedivers. I didn't fully understand what an ‘elite freediver’ was, but enthusiasm was not dampened. When we arrived in Croatia we were met by our local host (and freediving expert), Prof. Dujic Zeljko. We had a brief period to acclimate to his lab, and testing began the next day – there was no time for jetlag or to be a tourist.

On the first day of experimentation, I quickly learned what an elite freediver was. Foremost elite freedivers are athletes. These athletes are deeply committed to their sport, striving not only to optimize their performance but also to advance their understanding of the physiology and safety aspects of freediving through research. The sport of freediving operates no differently from any other conventional sports organization. Official freediving competitions and records are sanctioned under the International Association for the Development of Apnea, or AIDA for short (when abbreviated in French) (www.aidainternational.org). AIDA was formed in 1992, shortly after interest in freediving and record attempts ballooned from the movie *Big Blue*. As of writing, there are approximately 3000 athletes registered to compete in regular AIDA‐sanctioned competitions (this number does not include the hundreds of thousands of recreational freedivers). Athletes compete in 10 separate disciplines broadly characterized by vertical depth, horizontal swim distance (usually in a pool), and maximal breath hold duration. Each discipline involves a unique stressor, particularly when depth is involved (more on that later), but the unifying factor is a breath hold for an extended period.

The extended breath hold, and the accompanying physiological stress, is what initially brought us to study elite freedivers. On this trip (the first of many to follow), we were interested in the arterial blood gas profile and brain blood flow regulation. With breath holds averaging slightly longer than 5 min, the primary research finding from this trip was that at the termination of the breath hold, the cerebral oxygen delivery was maintained, despite the average end‐breath hold partial pressure of arterial oxygen of approximately 30 mmHg (Willie et al., 2015) – very close to 50% arterial oxygen saturation and the theoretical limit for consciousness (Nunn, 1987). The cerebral oxygen delivery was preserved through a proportional increase in brain blood flow that tightly compensated for the reductions in oxygen content. This trip was the tip of the iceberg, and unknown to me at the time, the *fons et origo* of my PhD trajectory and still a continued research interest now almost a decade past my PhD graduation. Through subsequent research expeditions with elite freedivers (see Figure 1 for a representative look at the instrumental set‐up with the freedivers), we gained insight into numerous fundamental questions, including the impact of hypercapnia and hypoxia on cerebral metabolism (Bain et al., 2016, Bain, Ainslie, Barak, et al., 2017), the ergogenic effect of beta blockers (substances banned by AIDA) on the maximal breath hold duration (Hoiland et al., 2017), the influence of lung volume (Bain, Barak, et al., 2017; Stembridge et al., 2017), the cerebral oxidative stress of maximal breath holds (Bailey et al., 2024 Bain, Ainslie, Hoiland, et al., 2018), and how the carotid body is involved with a maximal breath hold duration (Bain et al., 2015). A review of these findings and more related to the physiology of a maximal static breath hold (static meaning remaining stationary) is published in this journal (Bain, Drvis, et al., 2018).

**FIGURE 1:**
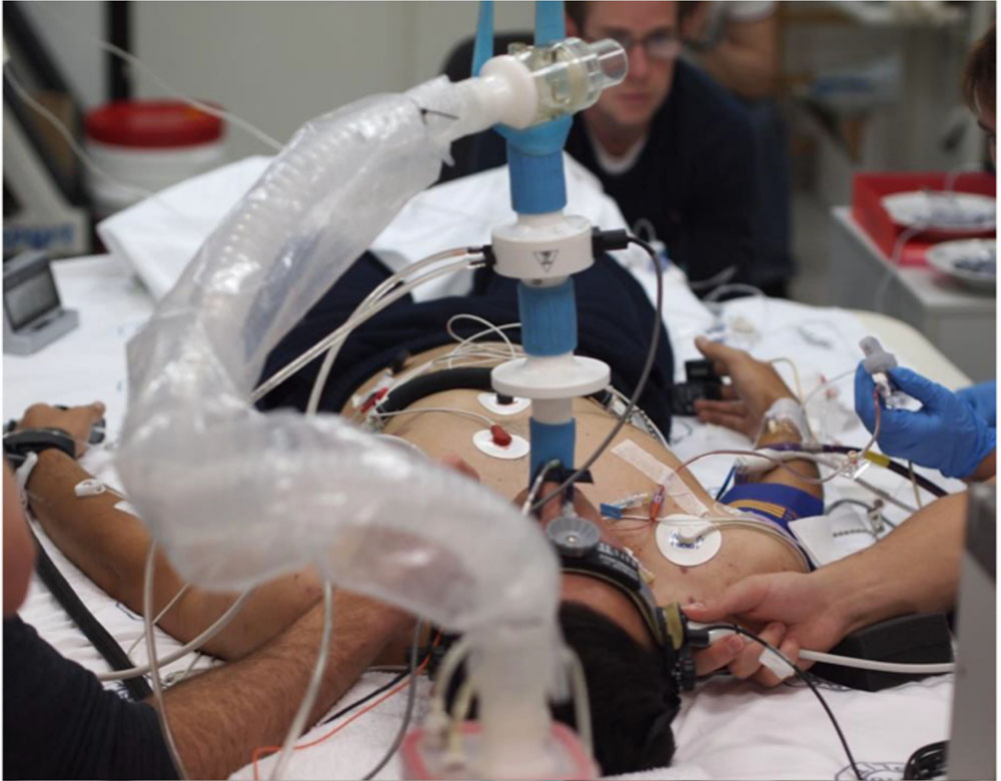
An elite freediver volunteering for research into the physiology of static apnoea.

These research trips (to date four in total spanning from 2013 to 2022) offered a wealth of academic experience and insight, and after completing my PhD I was considered an ‘expert’ in freediving physiology. But I hadn't done any freediving – or dedicated any convincing time to practicing extended breath holds myself. I felt like a fraud. I knew one day I'd need to give breath hold training a good attempt, and watching these athletes over the years sparked my inner sportsman's desire to test my own abilities. Thankfully, I was now armed with an arsenal of knowledge that I felt gave me a leg up.

I finally found some time to practice maximal breath holds before my summer holiday in Nova Scotia, in 2024. The motivation was garnered as I had convinced my brother to include two apnoea disciplines in our annual ‘feats of strength’ – a yearly event that moonlights as a gauge for who is physically deteriorating faster as we age. The two disciplines agreed upon were a maximal depth, closely resembling the Constant Weight No Fins (CNF) discipline in AIDA, and a static maximal breath hold. Unofficial Vegas odds had my brother as the favourite for the depth discipline, as he is a seasoned ocean swimmer and surfer, while I had a perceived advantage for the maximal static apnoea given my prior exposure to the sport. Another hotly debated consideration for the betting odds was that my brother was missing a spleen (after a splenectomy in 2003), which tentatively puts him at a disadvantage as the spleen acts as a reservoir for oxygen‐rich red blood cells, which are released during an apnoea and freediving (Inoue et al., 2013; Schagatay et al., 2001). Indeed, the Bajau divers in Southeast Asia, often referred to as ‘sea nomads’, evolved genetic alterations to have larger spleens as an adaptation to their freediving lifestyle (Ilardo et al., 2018).

To start training, I knew that breath hold exposure was key, that is, learning to become comfortable in the uncomfortable, or said differently, training your conscious brain (higher brain centres) to deprioritize the subconscious and mounting neural traffic. A prolonged breath hold evokes a powerful sympathetic stress response, with intensifying pressure from respiratory centres in the medulla, lung afferents and chemoreception (reviewed in Bain, Drvis, et al., 2018]) culminating in an increased drive to breathe. Occurring at around ∼3 min (albeit this is widely variable), the reflexive drive to breathe intensifies to the point where the diaphragm involuntarily contracts – usually referred to as an involuntary breathing movement (or IBM for short). This is where most naïve breath holders will break. However, through repeated exposures, you can learn to tolerate the IBMs and continue into the so‐called ‘struggle phase’ of the breath hold – the time in the breath hold before the IBMs is called the ‘easy phase’. Through training, upon each subsequent breath hold attempt, I was able to tolerate a few more IBMs before breaking. I was not close to the ‘elite’ level, where up to 100 or more IBMs may occur before breaking, but I was getting better. Interestingly, I was surprised to discover that the arrival of the IBMs began to provide a brief sense of dyspnoea relief. There are data suggesting IBMs may improve cerebral oxygenation through increases in venous return (Cross et al., 2013). Another untested theory is that IBMs momentarily reduce the mounting afferents from stretch receptors in the lung. Regardless, after confiding my personal findings with record‐breaking apnoeists from the National Croatian apnoea team, I am not alone in feeling a brief sense of dyspnoea relief with the onset of the IBMs. However, to experience any sense of transient ‘relief’ from the onset of the IBMs, it was important to stay relaxed.

This leads me to the most valuable aspect of my breath hold training – the mental component. Learning appropriate relaxation techniques was evident during my first exposure to the sport, where the best athletes entered a state of ‘Zen’ before any maximal apnoea attempt. Relaxation techniques serve two interconnected purposes. The first is that the perceived stress of the breath hold is attenuated, and the second is that it provides some oxygen conservation by reducing heart rate and therefore myocardial oxygen consumption. The mechanism at play is likely identical to how reducing central command during exercise can lower heart rate and blood pressure for a given absolute exercise intensity (Raven et al., 2002). Learning how to stay calm throughout a maximal apnoea, and becoming comfortable in the uncomfortable, was particularly important for me as I had very limited training time, and therefore the prospect for any measurable physiological adaptation was bleak. That is, the longer term (e.g., months to years) physiological adaptations to breath hold training may include a blunted ventilatory chemoreflex to hypoxia and hypercapnia (albeit there are conflicting data on this, which is fully reviewed in Bain, Drvis, et al. (2018)), larger lung volumes (mainly related to learning how to lung pack before a breath hold) (Ferretti & Costa, 2003), increased spleen size (Yang et al., 2022) and a heightened mammalian dive response (Ostrowski et al., 2012).

On our ‘competition’ day, we started with the maximal apnoea. To optimize the oxygen conserving effects of the mammalian dive reflex (e.g., bradycardia and central distribution of blood flow), this event would have ideally been performed in the water with the face submerged, as it is in AIDA sanctioned events. However, under guidance (authority) of our wives and family, the apnoea was performed on dry land, with no facial cooling. We each performed two submaximal preparatory breath holds a few minutes prior to the maximal attempt. The preparatory breath holds was a custom learned from the elite divers, which dramatically increases the maximal breath hold duration. These preparatory breath holds likely attenuate the initial ‘shock/stress’ response of an extended breath hold. While untested, there theoretically may also be some cerebrovascular conditioning effects.

Besides the inclusion of the preparatory submaximal breath holds, I provided no further advice to my brother. Because the breath holds were performed on dry land, there was no risk of shallow water blackout (Bart & Lau, 2025), and therefore I included some deep breathing/induced hypocapnia immediately before the maximal attempt. Knowing that the primary stress of an extended breath hold is from hypercapnia (elevated arterial $P_{CO_{2}}$), especially in the early stages before the blood oxy‐haemoglobin saturation begins to decline, starting the breath hold hypocapnic is advantageous.

In the end, the static apnoea discipline was an easy win for me – a respectable (for a novice) 4 min and 8 s. My brother mustered a measly 3 min and 2 s – notably a similar duration to my initial attempts before ‘training’. His breaking point probably coincided with the onset of IBMs (he had not learned how to hold the IBMs), as is common for motivated novice breathholders. The depth discipline was next.

Unlike the static maximal breath hold, I conducted no specific training for the depth competition. I knew this was a mistake, but I had also convinced myself that the dry land breath hold training was better than nothing. However, depth apnoea disciplines involve unique stressors compared to simple static apnoea. Swimming and buoyancy efficiency are essential to minimize oxygen use and production of metabolic CO_2_. Moreover, the pressure equalization techniques are essential to gain any sort of depth. Indeed, each 10 m descent under water is equivalent to adding an entire atmospheric pressure (∼760 mmHg). The air cavity squeeze felt while descending is explained by Boyle's law and is usually felt first in the air cavity of the middle ear (sinus pressure, especially when congested, can also be an issue). Failure to equalize the pressure can lead to barotrauma and damage to the eardrum and surrounding structures. Trained freedivers apply different techniques to quickly equalize inner ear pressure – the most common is the Frenzel manoeuvre (Wolber et al., 2021), but the best can do it with simple variations of a Valsalva manoeuvre and swallowing. The key is to equalize the inner ear cavity without losing lung volume. This is particularly important for elite divers who descend to extreme depths (the AIDA record for the CNF discipline is 290 m).

While the risk of eardrum barotrauma due to inadequate equalization was present, the likelihood of severe pulmonary barotrauma – commonly observed in elite divers reaching extreme depths (e.g., >200 m) – was not a concern. This was due to the relatively shallow conditions of our dive, with the bay's maximum depth being 30 m and our initial dives starting at just 10 m. However, the potential for shallow water blackout had to be considered. The cause of shallow water blackout is the rapid reduction in the partial pressure of arterial oxygen during the ascent, as barometric pressure is halving (compared to atmospheric) in the 10 m from surface level. As such, in AIDA‐sanctioned events, all dives are performed with safety divers on standby and medical personnel on the surface. For a dive to be considered successful, athletes must surface without showing any signs of impending or actual loss of consciousness, such as disorientation, unresponsiveness or motor control issues. Spotters closely monitor for these preliminary indicators of loss of consciousness to ensure the diver's safety. Unfortunately, insufficient funding for the annual ‘Feats of Strength’ competition did not allow for the implementation of such safety measures. Therefore, to minimize the risk of shallow water blackout we adhered to a strict no hyperventilation protocol before diving (elite divers also avoid hyperventilation before a depth dive). Indeed, the risk of shallow water blackout is heightened by prior surface hyperventilation before diving, as the drive to breathe from hypercapnia is abolished, and the cue to resurface is lost. We were also cognisant of the dangers and started cautiously.

When it came time for the dive, we dropped an anchor line attached to a buoy, with the first depth of approximately 10 m. The goal was to collect some sand from the bottom as proof of descent. The anchor would then move to deeper depths with successful attempts. My brother went first and easily came up with a handful of sand from 10 m below. The total apnoea time was probably less than 30 s.

My attempt, however, was abysmal. I had experience with pressure equalization while scuba diving, but doing it during a breath hold proved to be much more difficult. I descended approximately 4–5 m and paused, trying to equalize, but my efforts were unsuccessful, and I had to come up. In the CNF discipline, the rope cannot be used for assistance, making the dive uniquely challenging. The combination of maintaining buoyancy control, holding my breath and attempting to equalize proved overwhelming. This round of the competition ended almost as soon as it began.

In turn, my primary reflection on the ‘lived experience’ centres on the distinct physiological, physical and psychological challenges of static apnoea compared to a depth dive. Even for elite divers, a breath hold during the CNF discipline (Figure 2) typically lasts only 2–4 min due to its intense physical demands. (Conversely, the AIDA sanctioned static apnoea record duration is 11:35 min.) In my case, the extra energy and time spent attempting to equalize inner ear pressure ultimately became my undoing. My respectful maximal static apnoea record of 4 min and 8 s had no translation to the depth dive. On the other hand, while my brother had a poor maximal static apnoea time, his comfort level in the water, combined with more experience in pressure equalization, provided a clear path to victory. In the end, the unofficial Vegas betting odds were correct (the house always wins), and in many respects, as a freediving ‘expert’ I still feel like a fraud. However, as an academic, the lived experience provided some unexpected research questions that I now have a personal motivation to explore. First, what are the physiological impacts of the preparatory apnoeas that permit a longer maximal apnoea duration, and are they quantifiable? Additionally, what are the mechanisms by which the initial involuntary breathing movements provide a brief sense of dyspnoea relief (at least in me)? Are they quantifiable? While these questions may be challenging to tackle, I like to think I'll have more success in addressing them – certainly more than I had with my freediving attempt.

**FIGURE 2:**
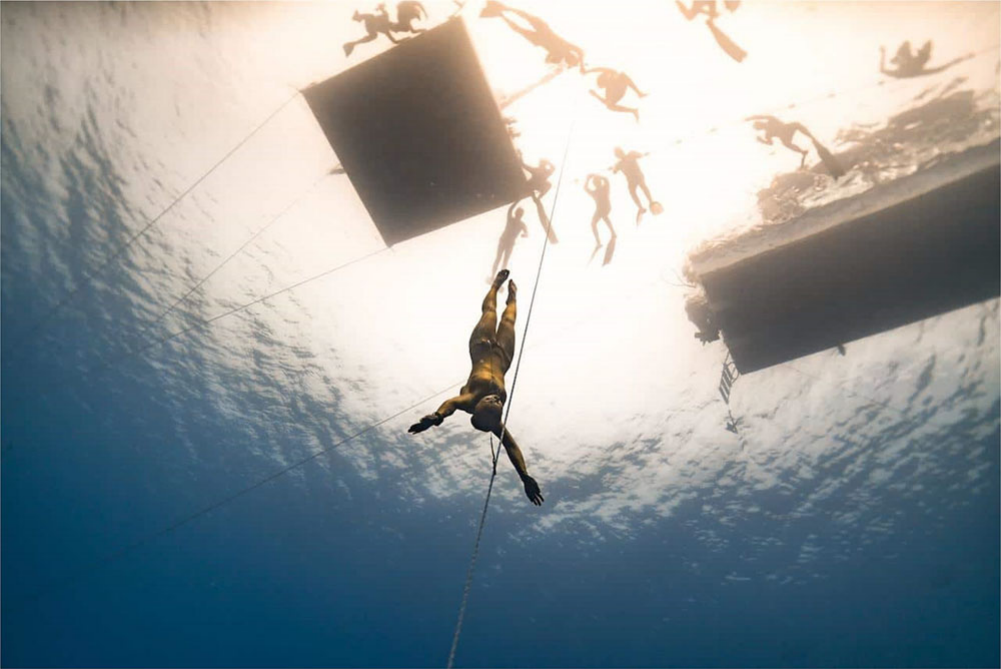
An elite freediver performing at the 2017 AIDA Individual Freediving Depth World Championships in the CNF discipline. Retrieved from www.deeperblue.com. Photo taken by Daan Verhoeven. CNF, Constant Weight No‐Fins.

## AUTHOR CONTRIBUTIONS

Sole author.

## CONFLICT OF INTEREST

None declared.

## FUNDING INFORMATION

No funding was received for this work.
